# Diversity and complexity in neural organoids

**DOI:** 10.1002/1873-3468.70329

**Published:** 2026-04-24

**Authors:** Ilaria Chiaradia, Madeline A. Lancaster

**Affiliations:** ^1^ Cell Biology Division MRC Laboratory of Molecular Biology Francis Crick Avenue Cambridge UK; ^2^ Cambridge Stem Cell Institute University of Cambridge UK

**Keywords:** assembloids, brain development, chimeroids, disease modelling, genetic diversity, neural organoids, pluripotent stem cells, tissue complexity

## Abstract

The complexity of human neurobiology is influenced by its heterogeneity, reflected in both cell type diversity as well as genetic variations between individuals. Neural organoids are 3D developing neural tissues designed to mimic development of the nervous system; however, most currently fail to capture this diversity. Recent advances have focused on increasing complexity through self‐organised multi‐region organoids, assembloids and chimeroids. Here, we discuss these approaches, as well as the remaining lack of genetic and ethnic diversity in neural organoid research, its impact on the generalizability of findings, and strategies to address this gap. We provide a flowchart to guide the experimenter towards the choice of model and discuss applications of neural organoids that benefit from complexity or reliability.

## Graphical abstract

Neural organoid research aims to expand genetic diversity on one side and increase tissue complexity on the other. Chimeroids integrate multiple donor genomes within single organoids. Self‐organising multi‐identity organoids, exogenous cell seeding, or enforced assembly of region‐specific organoids contribute to tissue complexity. These strategies broaden organoid applicability but may compromise reproducibility. We provide guidance between complexity and reductionism.

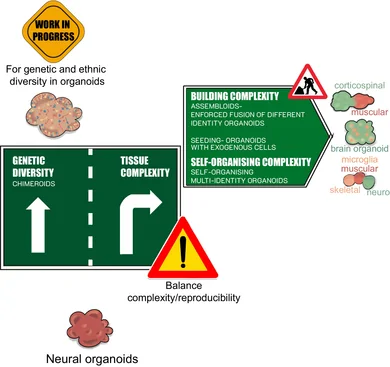

## Abbreviations

**ASD**, Autism spectrum disorder

**BBB**, Blood–brain barrier

**ChP**, Choroid plexus

**CRISPR**, Clustered regularly interspaced short palindromic repeats

**DA**, Dopaminergic

**EC**, Endothelial cell

**eQTL**, Expression quantitative trait locus

**ESC**, Embryonic stem cell

**ETV2**, ETS variant transcription factor 2

**FGF8**, Fibroblast growth factor 8

**hESC**, Human embryonic stem cell

**HLA**, Human leukocyte antigen

**HUVEC**, Human umbilical vein endothelial cell

**iPSC**, Induced pluripotent stem cell

**NMP**, Neuromesoderm precursor

**NSC**, Neural stem cell

**PSC**, Pluripotent stem cell

**RNA‐seq**, RNA sequencing

**RTT**, Rett syndrome

**SNP**, Single nucleotide polymorphism

**ZIKV**, Zika virus

Neural organoids are stem cell‐derived models of developing human brain tissue. Starting from embryonic stem cells (ESCs) or induced pluripotent stem cells (iPSCs), cell aggregates are grown in suspension to enable 3D tissue growth and development following self‐organising principles similar to brain development *in vivo* [1]. They can be grown in minimal conditions to allow mainly intrinsic developmental programmes of neural development, or in directive conditions with small molecules to promote specific neural identities [2]. The outcomes are different, and as discussed further below, the choice of what system to use should depend upon the question being asked. Intrinsic or unguided organoids favour multiple regional identities over homogeneity. Directed or guided organoids aim at higher inter‐sample reproducibility. Whereas the former may find applicability in fundamental research on brain evolution and growth, the latter are potentially better suited for higher throughput studies such as patient‐tailored disease modelling and drug screening.

Disease modelling and drug screening require high throughput and reliability to discern the pathological phenotype from inter‐sample variance, cell line effect and the influence of culturing conditions. Directed organoids [3] or single‐region spheroids [4, 5] are valuable alternatives to two‐dimensional neural cultures [6, 7] for tissue intrinsic questions, both in terms of complexity and reproducibility. Despite recent efforts, cell line effect and batch effect remain big obstacles for *in vitro* research. This has led to the use of a limited range of cell lines in experiments and the establishment of consensus cell lines for consistent results across users. The direct consequence is the limited representation of ethnic and genetic variation among organoids. Genetic differences veil differential susceptibilities to disease and responses to drugs. In an effort to study these genetic‐driven differences, chimeroids composed of multiple cell lines merged into a single organoid [8] or mosaic organoids with multiple neural disorder‐associated mutations [9] have been successfully generated, similar to research carried out on two‐dimensional cell villages [10]. Despite the growing number of patient‐derived iPSCs in organoid research, specific ethnicities remain underrepresented, thereby limiting the generalizability of findings.

Studying brain development and evolution demands models that closely resemble the entire *in vivo* brain. Brain function cannot be fully explained by summing the activity of individual brain regions and exploring one brain area at a time. Tissue complexity can be achieved either by leveraging intrinsic development of multiple tissue types in a single undirected organoid (self‐assembly [11, 12]), by assembling multiple region‐specific directed organoids (enforced assembly, also known as assembloids [13]), or by seeding non‐neural cell types onto neural organoids [14, 15]. This review examines the pursuit of genetic diversity and tissue complexity in 3D *in vitro* neural models, addressing the current state of the art, future directions and the trade‐off between complexity and analytical clarity.

## Genetic diversity in neural organoids

Genetic diversity is the backbone to human endurance and adaptability. Apart from identical twins, no two individuals are genetically identical, with an individual‐to‐individual genome diversity of 0.4% [16]. How much of this diversity is specific to brain‐enriched genes is not directly documented, but it can be extrapolated from genome‐wide regional gene expression analysis. Out of 15 157 protein‐coding genes detected in at least one region of the brain, 2587 genes show elevated expression in the brain compared with peripheral tissues [16]. Estimating an equal representation of the 0.4% individual genome diversity across different tissues, approximately 10 brain‐enriched genes would differ from one individual to another.

Importantly, human genetic diversity is not homogenously spread across populations. Populations of African ancestry were shown to have more genetic variation than any other ethnicity, including disease‐associated genes [17]. Despite efforts into the generation of Black African ancestry cell lines [18], most of the pluripotent stem cell (PSC) line repositories rely on Caucasian and Japanese donors [19]. A 2024 review of the major induced pluripotent stem cell (iPSC) biobanks extrapolated the following data: at CIRM, California Institute for Regenerative Medicine, approximately 71% of the annotated iPSC ethnicity is Caucasian; at the European Bank for Induced Pluripotent Stem Cells (EBiSC) ~82% of the cell lines with available data are described as Caucasian; at WiCell, where most of the cell lines have information about the ethnic origin, Caucasian accounts for ~67% of the iPSCs; RIKEN BRC in Kyoto stores cells with > 99% Japanese ancestry [20], whereas the Coriell Institute for medical research has 308 commercially available iPSC lines, out of which less than 20% have an annotated non‐white ethnicity [21]. This rooted inequality scratches the surface of a much deeper issue related to the applicability of the currently available iPSC lines. Regenerative medicine and human heterologous transplantation of PSC‐derived organoids are hampered by the risk of immune rejection due to the incompatibility of the cell surface antigens of the human leukocyte complex (HLA). The lesser the similarity between the donor and the acceptor HLAs, the higher are the chances of transplant rejection [22]. Individual HLA patient‐compatible or autologous transplantation are often out of reach due to the costs of the procedure. Conversely, a genetically diverse pool of PSCs would enable a greater match with the majority of human HLAs. For the generation of the first Brazilian‐endemic human embryonic stem cell (hESC) line, the authors looked for HLA compatibility in 22 hESC lines, including frequently used H1, H9, HUES7, across an HLA database with a large subset of the Brazilian population and concluded that the available cell lines only matched 0.011% of the Brazilian haplotypes [23]. Besides the limitations that poor genetic diversity across stem cell lines pose to regenerative medicine and stem cell therapy, this also hampers novel discoveries of disease‐associated allele variations. Small nucleotide polymorphisms might be pathology‐linked in one genetic background but not in others.

In general, organoid research suffers from a lack of genetic diversity both in terms of ethnicity and population genetics. The main challenge is currently the mitigation of the variability across experiments from different PSC lines. This unfortunately overshadows individual‐to‐individual intrinsic biological differences and hampers the generalizability of findings and novel medical applications. On the other hand, the identification of a consensus cell line protects from cell line‐to‐cell line variability attributable to the age, sex of the PSC donor, to the reprogramming protocol used, and to the culture conditions. Differences between donor individuals explain up to ~46% of gene expression variation and up to ~23% of differences in cellular morphology across 711 iPSC lines from 301 healthy individuals [24]. Immunostaining for markers of pluripotency displays a donor variance of ~21–46% only exceeded by the assay batch effect [24, 25]. These studies bring up the question of whether using multiple genetic backgrounds for a given experiment could result in novel insights or rather nonspecific effects due to cell line variance. What is certain is that different iPSC lines should be cultured following the same protocol. Cell lines from different ethnic groups and laboratories worldwide were shown to acquire chromosomal changes, despite remaining karyotypically normal, across prolonged times in culture. Regardless of the cell line tested, cells showed a non‐random gain of genes in chromosomes 1, 12, 17, 20 that confers a growth advantage [25].

In an effort to standardise the field, the iPSC KOLF2.1 J has been identified as a cell line with better yield of neural differentiation and has been suggested as the standard for broad use across different laboratories [26]. Such a standard can safeguard the interpretability of data and can be used to explore the impact of genetic and environmental perturbations in a highly controlled fashion. Introducing specific mutations in a genetically controlled background enables the mechanistic investigation of gene function without the confounding factor of cis and trans regulators that might work differently in different genetics. Such so‐called isogenic lines are an effective and complementary alternative to patient‐derived iPSCs. Multiplexed CRISPR perturbations on hESCs have enabled the generation of individual organoids with up to 36 autism spectrum disorder (ASD) high‐risk mutations, each carried by individual cell clones of a mosaic brain organoid [9]. This multi‐isogenic line organoid minimises the batch effect that might arise from the manipulation of different organoids, each with a different ASD‐linked mutation. However, the background cell line used was a single line, the hESC H9 line. Thus, the influence that different genetic backgrounds might have on the pathophysiology of each mutation is unknown. Likewise, the KOLF2.1 J is of European ancestry, thus failing to capture ethnic genetic diversity. Ideally, in the future, isogenic mutations could be introduced in several genetically diverse cell lines to capture population differences.

Recently, a major effort was published that has integrated several existing transcriptomic data from human neural organoid studies [27]. This dataset covers 36 RNA‐seq experiments from research articles published between 2017 and 2024 and shows a total of 7 hESCs and an estimated 128 iPSCs being used across 8 years of research progress in the field (Table 1). This represents a significant effort towards a broader coverage of population genetics, although concerns remain regarding the unbalanced ethnic representation. Recent advances have moved in the direction of integrating a greater genetic diversity in individual organoids through cell pools, chimeroids, and mosaic organoids (Fig. 1).

**Table 1:** PSCs used in neural organoids.

Publications	Cell lines used
Kanton, 2019 [28]	H9 hESC; 409b2, Sc102a1, Hoik1, Kucg2, Sojd3, Wibj2 hiPSCs
Fleck, 2023 [29]	H1, H9, HES3 hESCs; 409B2, B7, HOIK1, KUCG2, WIBJ2, WTC hiPSCs
Sawada, 2020 [30]	DT1_A, DT1_U, C1, C2, C3, C4 hiPSCs
Uzquiano, 2022 [31]	H1, HUES66 hESCs; GM08330, PGP1, Mito210, 11a hiPSCs
Vertesy, 2022 [32]	H9 hESC; SCCF – 177, SCCF – 178, HPSI0114i‐rozh_5 hiPSCs
Pellegrini, 2020 [33]	H9, H1 hESCs
Kelava, 2022 [34]	H9, H1 hESCs
Velasco, 2019 [35]	HUES66 hESC; GM08330, PGP1 hiPSCs
Bhaduri, 2020 [36]	H28126, 13 234, WTC10 hiPSCs
Paulsen, 2022 [37]	H1, HUES66 hESCs; Mito210, Mito294, PGP1, GM08330 hiPSCs
Birey, 2017 [5]	H9 hESC; 6 control hiPSCs, 7 hiPSCs from three subjects with TS, H20961
Khan, 2020 [38]	43 hiPSCs from 15 patients with 22q11.2 deletion and 15 controls
Sloan, 2017 [39]	8858‐1, 8858‐3, 1205‐4, 2242‐1 hiPSCs
Trujillo, 2019 [40]	C1, C2, C3 (Nageshappa et al., 2016), WT126, WT33, WT83 (Tang et al., 2016) hiPSCs
Yoon, 2019 [41]	15 hiPSCs from 13 healthy subjects (including H20961, LMNB1‐mEGFP (AICS‐0013), TUBA1B‐mEGFP (AICS‐0012))
Bowles, 2021 [42]	GIH6‐1‐C1, GIH7‐C2, ND32951A.15 hiPSCs
Qian, 2020 [43]	C1‐2, C3‐1, D2‐1, D3‐2 hiPSCs
Miura, 2020 [44]	5 control hiPSCs from 5 healthy subjects, 3 hiPSCs from 3 patients with 22q13.3 DS
Samarasinghe, 2021 [45]	H9 hESC; Rett hiPSCs GM07982, GM17567 and the respective control hiPSCs
Huang, 2021 [46]	C3, C65, PWS hiPSCs
Xiang, 2019 [47]	H1, HES‐3 hESCs
Fiorenzano, 2021 [48]	RC17, HS999, HS1001, H9 hESCs
Atamian, 2024 [49]	H9 hESC; 11a, D2 hiPSCs
Andersen, 2020 [50]	9 hiPSCs from eight healthy subjects
Marton, 2019 [51]	7 hiPSCs from 7 healthy subjects, H20961 hiPSC

hESCs: H1, H9, HES3, HUES66, RC17, HS999, HS1001.

hiPSC lines: 409B2, SC102a1, Hoik1, KUCG2, Sojd3, WIBJ2, B7, WTC, DT1_A, DT1_U, C1, C2, C3, C4, GM08330, PGP1, Mito210, 11a, SCCF–177, SCCF–178, HPSI0114i‐rozh_5, Mito294, H20961, 13 234, H28126, WTC10, 2242–1, 1205–4, 8858–3, 8858–1, C1, C2, C3, WT126, WT33, WT83, LMNB1‐mEGFP (AICS‐0013), TUBA1B‐mEGFP (AICS‐0012), GIH6‐1‐C1, GIH7‐C2, ND32951A.15, C1‐2, C3‐1, D2‐1, D3‐2, GM07982 control line, GM17567 control line, GM07982, GM17567, C65, PWS, D2, 15 patients with 22q11.2 deletion + 15 controls hiPSCs (Khan, 2020 [38]), 5 control + 3 patient hiPSCs from 22q13.3 DS (Miura, 2020 [44]), 9 hiPSCs from 8 healthy subjects (Andersen, 2020 [50]), 7 hiPSCs from 7 healthy subjects (Marton, 2019 [51]), 15 hiPSCs from 13 healthy subjects (three already counted above) (Yoon, 2019 [41]), 6 control hiPSCs, 7 hiPSCs from three subjects with TS (Birey, 2017 [5]).

**Fig. 1:**
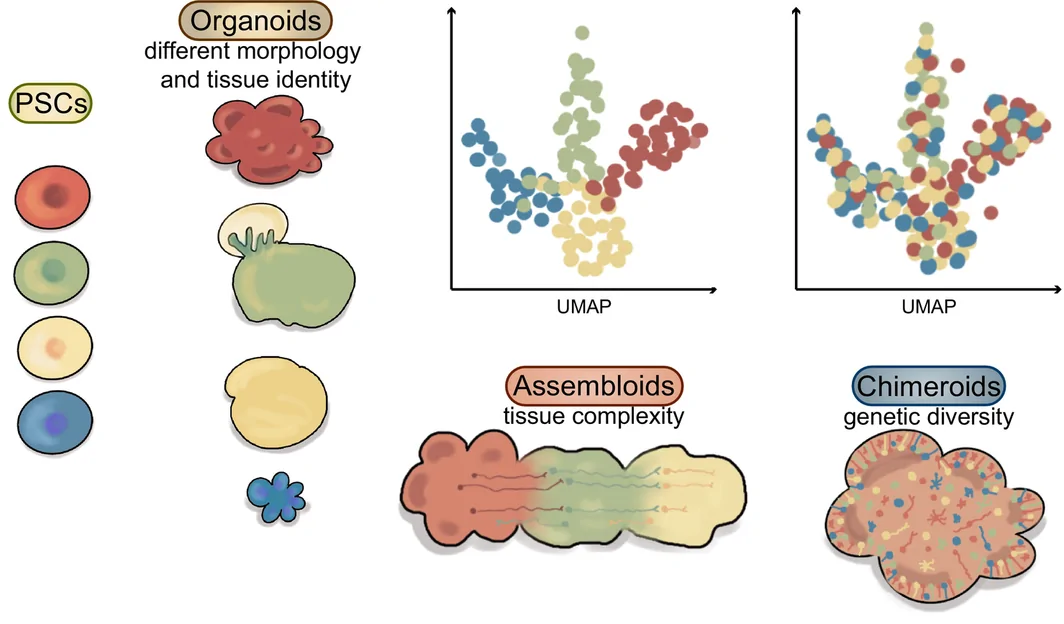
Genetic and tissue diversity in neural organoids. Pluripotent stem cells (PSCs), including embryonic stem cells (ESCs) and induced pluripotent stem cells (iPSCs), upon neural differentiation give rise to neural organoids with varying tissue identity, size, and morphology. This variation results in distinct cellular profiles across different cell lines. Studies on single cell line organoid restrict the genetic diversity represented across the population. Chimeroids address genetic diversity by incorporating multiple PSC lines within a single organoid. When donor cells are pooled at the neural stem cell (NSC) stage, homogeneous cell type representation can be achieved. Assembloids address tissue complexity by fusing region‐specific organoids to model complex neural circuitry.

## It takes a village: Chimeroids address genetic diversity

Not all cell lines behave equally upon differentiation. Many underlying factors, including the donor's age, the reprogramming protocol, sex, and culturing conditions, all contribute to different outcomes. Hundreds of human iPSCs from the Human Induced Pluripotent Stem Cell Initiative (HipSci) were directed to definitive endoderm [52] and dopaminergic neurons (DA), as well as cerebral organoids [53] through pooled differentiation assays of multiple lines in the same experiment. Downstream analysis of expression quantitative trait loci (eQTL)—genetic variants that explain variation in gene expression between individuals—ruled out 13% of the 812 analysed hiPSCs for their poor differentiation outcome to DA neurons. Importantly, several of these eQTL colocalized with disease traits. Consistently, several genetic variants affecting differentiation of PSCs were linked to neurodevelopmental disorder candidate genes [10, 54]. This advocates for a careful analysis of the pathological phenotype based on its genetics.

Cell line pooling is often referred to as cell villages [10]. The technique maximises the readout of expression variance from multiple genetic backgrounds with the aim of uncovering cis‐acting regulators in a scenario where environmental trans‐acting elements should be homogenous across the culture. Census‐seq and Dropulation are popular analyses to deconvolute the multiplexed village RNA‐seq dataset and to dig out individual single nucleotide polymorphisms (SNPs) associated with expression variance in nearby genes while ensuring that inter‐line gene expression is not affected by village culturing conditions [55]. Village culture can be used with CRISPR‐Cas9 edited isogenic lines to study multiple mutations at once. When cells with different autism spectrum disorder (ASD) high‐confidence mutations were pooled, the assay revealed a delayed neurogenesis during prefrontal cortex 2D induced differentiation for some lines, and a premature neurogenesis for other lines [56]. Most of the expression variance derived from the asynchronous differentiation of cell lines, rather than from their pluripotent stage, suggesting that while pluripotency depends on the activation of few conserved pluripotency genes, differentiation instead is orchestrated by a plethora of cell‐intrinsic factors highly susceptible to interindividual differences. In a different study, cell line differences during differentiation into neural lineage were able to explain up to 99.4% differential vulnerability to Zika virus infection across a multi‐donor neural progenitor village [10].

The village approach has also been applied to 3D brain organoid differentiation, also referred to as multiplexed organoids, mosaic organoids, or multi‐donor chimeroids. The term multiplexed organoids refers to pooling different cells during organoid generation or just before single cell RNA‐seq [57]. On the other hand, the term mosaic organoids refers to CRISPR‐Cas9 induced mutations through different guide RNAs on the same cell line [9]. Finally, chimeroids refer to multiple hiPSCs mixed post neural induction [8] (Fig. 1). Similar to what was described for 2D cultures, mosaic organoids with cells harbouring loss‐of‐function mutations for a combination of ASD risk genes revealed regions of the organoid with excitatory progenitor cell depletion and others with inhibitory progenitor cell enrichment [9]. Different genetic perturbations led to different cell behaviour, reflecting the variety of genetically linked ASD phenotypes observed both *in vivo* and *in vitro*.

Selective growth advantage is a dangerous bias in merged cultures, especially when dealing with PSCs [53, 57]. Fast‐paced proliferating cells might take over the culture, resulting in a skewed cell line representation. On top of differences in growth rate [57], different cell lines segregate into clones giving rise to separate organoid areas with poor intermingling [58]. Clonality is reduced when multi‐donor neural stem cells (NSCs) are pooled together [8], instead of PSCs, thus overcoming the need for a balanced iPSCs selection. This is the approach used in brain chimeroids to investigate interindividual differences in the susceptibility to neurotoxic substances, such as valproic acid [8].

Chimeroids and cell village approaches represent an unprecedented leap in organoid scalability. The method enables the high throughput investigation of the cross‐talk between genetic elements and gene expression, and importantly, it addresses the technical issue of batch effects, since all the cells are part of the same organoid batch. Despite the necessity for careful controls for cell line representation, selective growth advantage, and clonality, multi‐donor organoids leverage organoid complexity and offer great promises for disease modelling across a diversity of ethnic and genetic backgrounds.

## Building tissue complexity: Self‐assembly and enforced assembly

Tissue complexity is achieved through temporally and spatially orchestrated cell fate commitment. To be defined as such, a tissue must break symmetry, have internal organisers, coordinated interaction between different cell types, and a clear 3D architecture. During development, self‐assembly of multiple cell types, often from different germ layers, contributes to tissue formation. *In vitro*, the coexistence of multi‐cell layer derivatives remains a challenge. For instance, the mesoderm master regulator brachyury promotes axial mesoderm differentiation by inhibiting spinal cord fate. Conversely, cells positive for neural marker SOX2 downregulate brachyury and become spinal cord [59]. This mutual exclusivity, however, is only a matter of time. There is a timeframe in development when few epiblast cells co‐express SOX2 and brachyury in neuromesoderm precursor cells (NMPs) [60]. These poised or bivalent precursors can be leveraged *in vitro* to generate self‐assembled multi‐tissue organoids. This is the case for 3D neuromusculoskeletal organoids, harbouring spinal cord identity together with skeleton and skeletal muscles [11], all co‐occurring under the same culturing conditions and resulting in motor neurons functionally innervating paraxial muscles (Fig. 2).

**Fig. 2:**
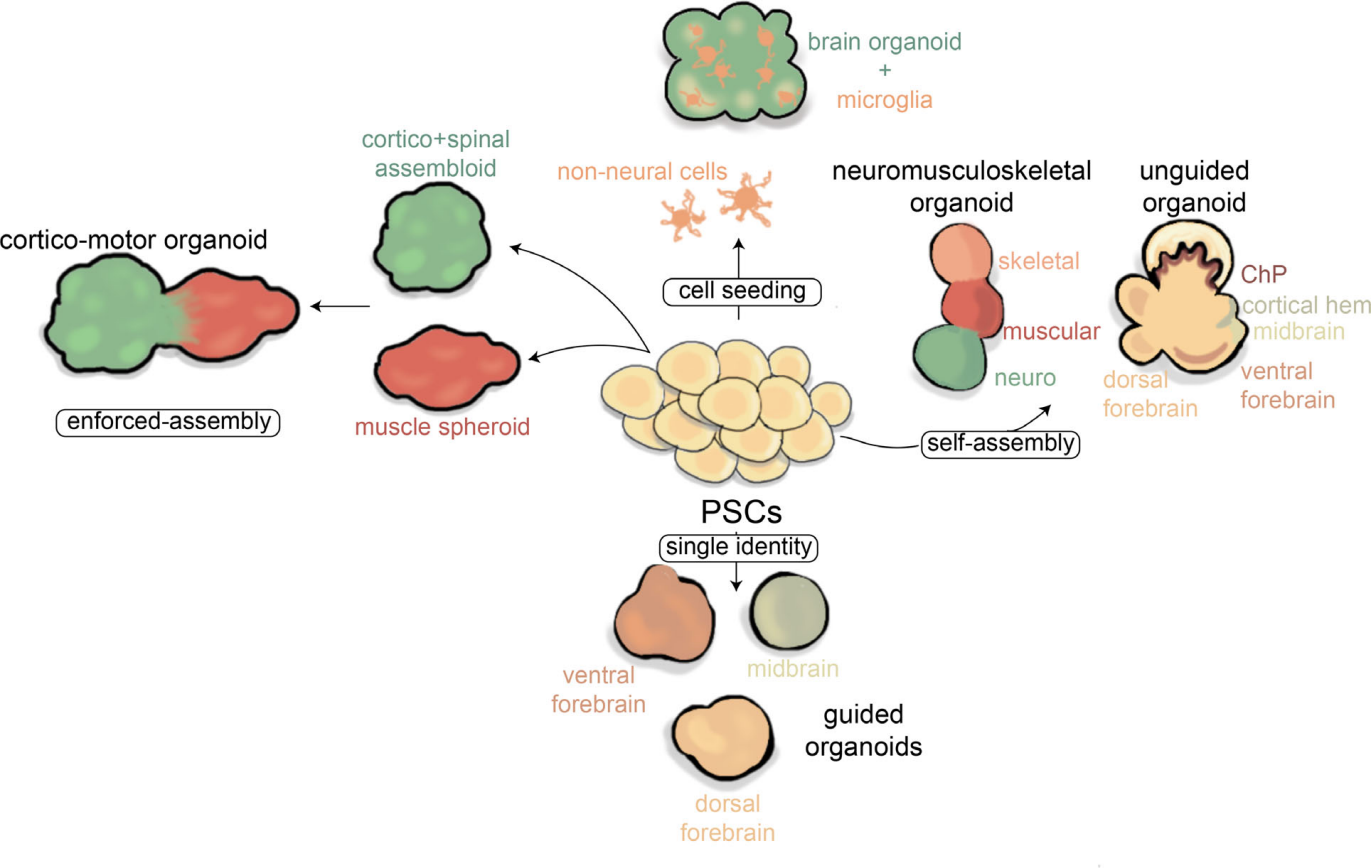
Strategies to achieve tissue complexity in neural organoids.Tissue complexity can be achieved through spontaneous differentiation and self‐assembly (right): pluripotent stem cells (PSCs) undergo spontaneous multi‐lineage differentiation and organ self‐assembly. 3D neuromusculoskeletal organoids, harbouring spinal cord identity together with skeleton and skeletal muscles, derive from neuromesoderm precursors obtained from PSCs [11]. Spontaneous development of diverse neural and non‐neural tissue occurs in unguided brain organoids where ventral and dorsal forebrain coexist with choroid plexus (ChP), cortical hem, midbrain, hindbrain and mesoderm‐like cells [61, 62]. Enforced assembly of multiple tissue organoids (assembloids) (left): Fusion of distinct organoid types (e.g. cortical, spinal and skeletal muscle organoids) creates cortico‐motor assembloids that mimic high‐order tissue interactions [50]. In this case, organoid fusion is obtained on already‐differentiated individual organoids. Cell seeding and extrinsic assembly (top): mesodermal cells derived from PSCs, such as microglia, are seeded on brain organoids to achieve neural‐non neural cell interactions and increase the complexity of the tissue [14]. Guided differentiation to single identity tissue (bottom): PSCs are directed to single identity organoids through guided protocols [3, 44, 49, 63, 64, 65]. This case represents the lowest tissue complexity achievable in 3D.

Similarly, multi‐lineage human intestinal organoids with epithelial cells, mesoderm‐derived mesenchymal cells, enteric neurons, endothelial cells and smooth muscle were shown to self‐develop from iPSCs treated with epiregulin, a polyvalent cell fate inducer found in enteric stem cell niches [12]. Gastrointestinal organoids, on the other hand, are commonly obtained from the guided assembly of iPSC‐derived enteric neuroglia, mesenchyme, and epithelial precursors grown independently. Three‐germ layer stomach assemblies were shown to functionally integrate muscle and epithelium—with peristaltic‐like contractions—and enteric neurons promoting gland development [66]. Amid the challenge of obtaining high reproducibility, self‐assembly strategies mimic more faithfully *in vivo* organ development than enforced assembly of separate identity organoids or cells.

Self‐assembly of neuronal, neural, and non‐neural cell types occurs spontaneously in unpatterned neural organoids [61, 62]. Mesoderm‐like cells, non‐neural and their derivatives—endothelial cells, microglia—were found in RNA‐seq datasets from unguided organoids [28, 62]. However, the representation of extra‐CNS‐derived cells remains scarce, and they display limited functional maturation. Alternatively, neural and non‐neural coexistence can be enforced by seeding neural organoids with non‐neuroectoderm cell types (Fig. 2). Microglia‐like cells from iPSCs were seeded on brain organoids, where they integrated into the tissue by chemotaxis and showed functional activation upon tissue injury [14]. Exogenous microglia seeding enabled a more robust representation across the tissue when compared to sparse innately developed microglia [67].

Seeding of exogenous cell types and functional integration in the organoid was also observed in brain organoids with vascular cell types. Human umbilical vein endothelial cells (HUVECs), which are endothelial cells (ECs) from the umbilical cord, were co‐cultured with PSCs and further grown in neural induction medium. ECs formed vessel‐like tubes invading and positioning adjacent to neural progenitor zones. This vasculature‐like net was shown to promote an increase in thickness of proliferative layers together with the increase in size of the whole organoid and the decrease in overall cell death [15]. In this instance, the intrinsic obstacle of trying to co‐differentiate very different cell types was avoided by using already‐differentiated ECs. In an alternative approach, ECs and neurons were obtained from PSCs under the same media conditions by using ETV2‐overexpression in a subset of cells to induce ECs in organoids. ETV2 is a cell master regulator that triggered the differentiation into ECs. Organoids with such vascular cells displayed a similar composition and architecture to non‐vascularised brain organoids but enhanced functional maturation and blood–brain‐barrier‐like structures [68]. Semi‐vascularised organoids or organ buds were also obtained through the induced fusion of vasculature‐like organoids and brain organoids [69] or mesenchymal and endothelial condensates with embryonic/adult‐derived brain tissue [70]. Fusion of brain organoids and vascular networks was enforced after both tissues were terminally differentiated. As expected, the assembly required neural supportive medium with the addition of vascular endothelium growth factor [69]. Nevertheless, this protocol did not impact the development of the organoid that instead showed increased numbers of neural progenitors, microglia, and blood–brain‐barrier identity. Despite similar end‐points, post‐differentiation enforced assembly of vascular and brain organoids displayed a less widespread vasculature, with no vessels invading the proliferative neural layer as compared to ETV2‐induced vasculature [68].

Overall, building tissue complexity often means overriding the tight temporal and spatial barriers of development. Complexity can be obtained through self‐assembly of multiple‐lineage cell types from a common progenitor, cell co‐culture or seeding, and enforced assembly of already‐differentiated tissues (Fig. 2). The first relies on the same culture conditions for different cell outcomes. The second and the third often require intermediate culture media for different cell fates. On a scale from reproducibility to physiological relevance, self‐assembled tissues outscore assemblies in matching *in vivo* tissue genesis but may exhibit lower consistency.

Timed tissue fate acquisition is a challenge for the coexistence of a variety of neural tissues. Individual organoids exist for dorsal and ventral forebrain [4, 5], thalamus [47], midbrain [3], cerebellum [49, 63], striatum [44], brainstem [64] and spinal cord [65] but they rarely develop together all at once (Fig. 2). The timed acquisition of dorso‐ventral and rostro‐caudal neural tube identity plays a role in this. The neural tube closes from the midline to the most rostral and caudal ends, with the anterior region being the last to mature [71]. The anterior–posterior axis is already established in the epiblast pre‐gastrulation, where OTX2 expression predicts cells that will turn into anterior neural tube [6, 72]. This early commitment to anterior fate, and the spontaneous differentiation of PSCs *in vitro* towards anterior neuroectoderm [73], suggests that self‐developing of brain and spinal cord identity in the same organoid and with the same neural induction protocol might not be feasible. Hence, other approaches have been developed to build complex tissues with multi‐regional specification and function: assembloids.

## When reductionism is enough, when complexity is needed

Assembloids consist of fused individual organoids with different identities (Figs. 1, 2). For two‐part assembloids, the time required for fusion is 2–3 days in culture [74], whereas for three to four‐part assembloids, it extends to one week [75]. Fusion represents a more stable and long‐lasting interaction of the components compared to more classical 2D co‐cultures [50]. What they have in common is the lack of spatial axis. Attempts to obtain a cortical organoid with axis specification involved fusing a FGF8‐secreting organoid—resembling the forebrain organiser—and a long tail of cortical tissue. The result was an assembloid with the proximal segment expressing markers of pre‐frontal cortex while the medial/distal area showed temporal cortex signature. Individual spherical organoids and spheroids are perhaps too small to achieve such a spatial gradient [76].

Not all assembloids are engineered in such a way. Assembloids of neural sensory pathway, where somatosensory, spinal, diencephalic, and cortical organoids are fused, display oriented functional activity with unidirectional stimuli travelling from the somatosensory to the cortical end but without axial coordinates [75]. Similarly, a loop circuit assembloid, made from adjacent cortical, striatal, mesencephalic and diencephalic organoids, is a four‐part loop with synchronised neural activity but no spatial axis [77]. Assembloids can indeed be bioprinted according to the experimenter design to maximise multi‐tissue interactions [74]. The current absence of axis organisation in assembloids will hopefully be addressed in future studies to resemble the higher order tissue structure achieved in stem‐cell‐derived embryo models *in vitro* [67, 78, 79].

On the quest to complexity, reductionist approaches, such as 2D neural cultures or 3D single‐region organoids, are still relevant models for disease modelling, drug screening, cytotoxicity assays, and for the investigation of cell‐autonomous phenotypes. Complementarily, assembloids or organoid‐on‐chip technologies [80] are crucial for functional studies on neural circuits and multi‐cell interactions (Fig. 3). Brain organoids were successfully used to study the tropism of ZIKV for neural progenitors in the forebrain, which identified impaired proliferation and a microcephaly‐like phenotype [85]. High‐throughput drug screening has identified promising antiviral candidates [86]. Brain organoids are effective and sufficient to study cell‐autonomous defects associated with individual mutations. This is the case for many ASD‐related mutations resulting in aberrant neural progenitor proliferation or neuronal differentiation [61, 87, 88]. 2D *in vitro* neural cultures are often enough to address the pathophysiology of individual genes. Patient iPSC‐derived Timothy syndrome Ca_v_1.2 mutated neural precursors and neurons revealed cell‐intrinsic changes in calcium activity, changes in gene expression, unbalanced production of noradrenergic and serotoninergic neurons, and a decrease in callosal projection neurons [89]. However, it was only through the development of ventral forebrain‐dorsal forebrain assembloids that further disease‐related neural circuitry alterations were uncovered *in vitro*. Birey et al. reported more frequent but shorter migration of interneurons from ventral towards dorsal regions of the assembloids, a defect that was restored by reducing the activity of the calcium channel targeted by the syndromic mutation [5]. The establishment of such cortical microcircuitry in assembloids enabled the characterisation of a functional phenotype that regular organoids failed to show. Similarly, Rett syndrome (RTT) is a neurodevelopmental disorder with a plethora of pathological phenotypes. Dorsal forebrain organoids from RTT patient‐derived iPSCs showed Notch signalling‐mediated premature deep‐layer neuron differentiation and reduced numbers of progenitor cells, although morphological features of the organoids generated lacked complexity. Ventral RTT organoids displayed a reduced expression of ventral progenitor markers. When assembled, the aberrant migration of interneurons from ventral to dorsal was revealed, with RTT interneurons from ventral travelling a shorter distance compared to healthy controls [90].

**Fig. 3:**
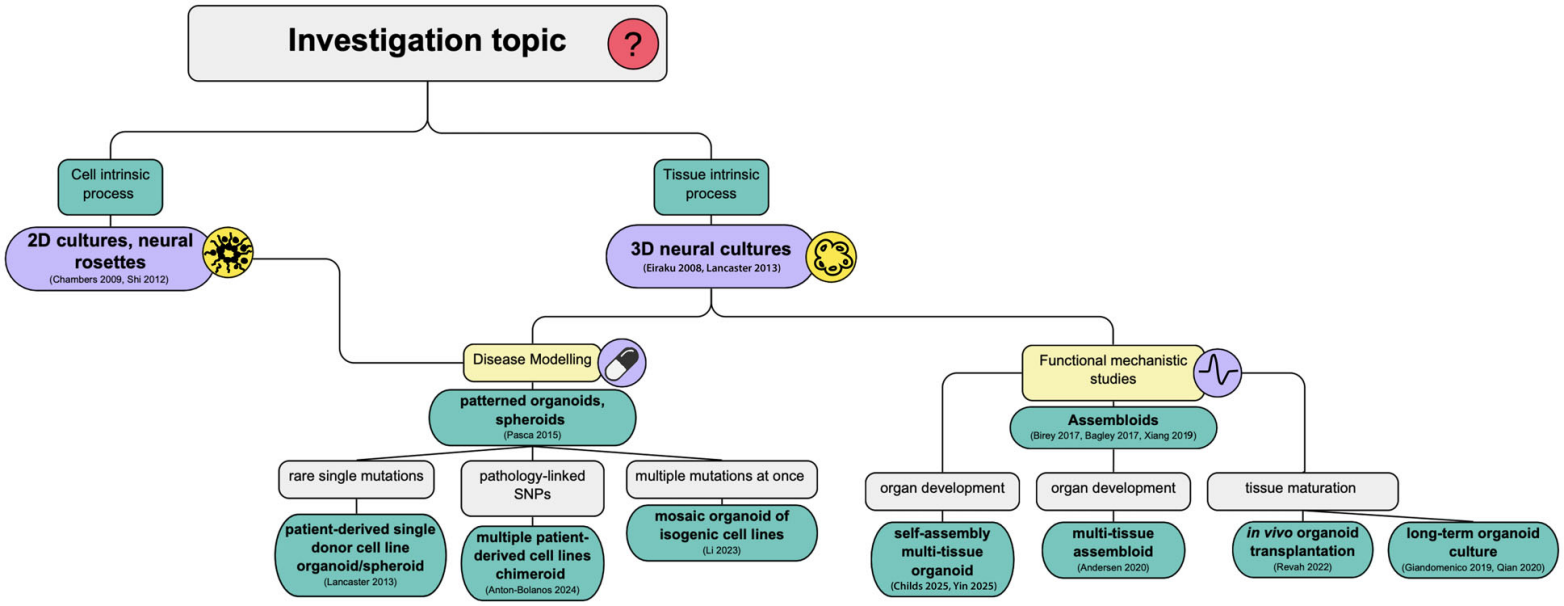
Flowchart for selecting an appropriate *in vitro* neural model based on research goals. A schematic flowchart guiding the choice of neural organoid models in accordance with the specific focus of investigation. Self‐assembly multi‐tissue organoids differ from multi‐tissue assembloids for the intrinsic self‐differentiation and self‐organisation of multiple cell and tissue types from the same pluripotent progenitor, often under the same culturing conditions. Refs: [4, 5, 6, 7, 8, 9, 11, 12, 43, 47, 50, 61, 81, 82, 83, 84].

Complexity is often coupled to reproducibility issues. The higher the number of components assembled, or cell types represented, the higher the failure rate (Fig. 4). Successful fusion is either assessed qualitatively through morphological integration or through the quantitative readout of the activity of the newly formed microcircuit. For a two‐unit assembloid, such as dorsal‐ventral forebrain assembly, 90% fusion success was shown across approximately 100 trials [4]. In cortico‐motor assembloids of human cortical, spinal, and skeletal muscle spheroids, the success rate of contracting muscle tissue upon cortical stimulation was 81% [50]. Instead, self‐assembly neuromusculoskeletal tri‐tissue organoids showed successful differentiation and self‐organisation into the three distinct domains in 93% of attempts with the H9 line [11]. This highlights a better yield for self‐assembly strategies compared to enforced assembloids. However, cell line variability accounts for major differences here, with a success rate going from 93% to 88% for the H1 cell line and 86.3% for the RC01001A hiPSC line. Four‐part assembloids of human ascending neural sensory pathway reported the formation of assemblies suitable for functional assays in 87.7% of cases [75]. Sample‐to‐sample reproducibility reaches the lowest value with loop circuit assembloids of cortical, striatal, mesencephalic and diencephalic organoids. Here the desired morphology, correct fusion and stable assembly were achieved in only 60% of trials [77] (Fig. 4).

**Fig. 4:**
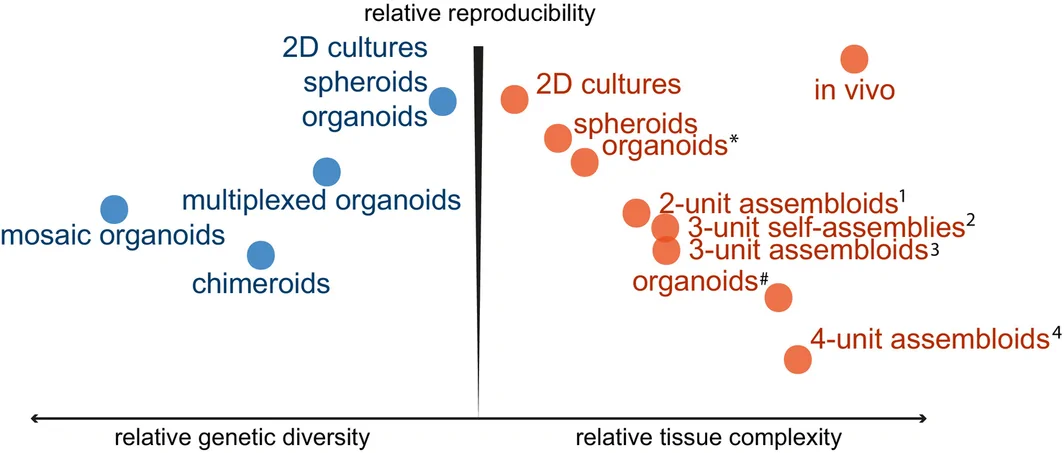
Balancing complexity and reproducibility in neural models. Conceptual not‐to‐scale relative positioning of 2D cultures, spheroids, organoids, self‐assembly, and multi‐unit assembloids across genetic diversity, reproducibility and tissue complexity. Tissue complexity often comes at the cost of reproducibility. Assembloids, which model multi‐tissue interactions, typically reduce experimental reproducibility—reproducibility decreases as more neural components are assembled. Single cell line organoids, spheroids and 2D cultures are limited in genetic diversity. In contrast, chimeroids, mosaic organoids, and multiplexed organoids support high‐throughput screening across multiple PSC lines retaining an overall good reproducibility across experiments. These approaches enable the analysis of disease phenotypes in different genetic backgrounds, SNP‐associated traits, thereby addressing genetic diversity. Organoids display different degrees of tissue complexity and reproducibility depending on the protocol. Self‐organising organoids (#) from unguided protocols can achieve greater complexity than assembloids but less reproducibility [61, 62]. Instead, single‐tissue directed organoids (*) display lower complexity and high reproducibility [3, 44, 49, 63, 64, 65], similar to spheroids [82]. ^1^Representative success rate of the assembly: 90% [4]. ^2^Representative success rate of the assembly: 86.3–93% [11]. ^3^Representative success rate of the assembly: 81% [50]. ^4^Representative success rate of the assembly: 60–87.7% [75, 77].

This success rate remains impressive, especially given the inherent variability of organoids and the complexity of the neural circuitry the authors aimed to recreate. To further enhance assembloid reproducibility, future efforts could focus on implementing quality control of pluripotent stem cells to start with—assessing their genetic stability and differentiation potential—conducting parallel experiments with multiple cell lines, and introducing defined axial patterning via tissue organisers or signalling centres. While these strategies may be more technically demanding than allowing self‐organising multi‐tissue organoids to develop autonomously, they might offer greater control and reliability for assembloid cultures. In conclusion, complexity may not be necessary for every application. Its use should be tailored to the specific biological question, to balance cost effectiveness and rigour for increasingly comprehensive *in vitro* models.

## Conclusion

After a decade of organoid research, two major considerations emerge: how to obtain the genetic diversity of the human population *in vitro* and how to mimic *in vivo* multi‐tissue complexity. Whereas case–control studies in patients often require hundreds to thousands of individuals to validate the observations, *in vitro* studies on human organoids are performed on a few cell lines with limited genetic and ethnic coverage. This approximation is generally accepted given the difficulty to obtain and handle multiple cell lines for the same experiment. Expanding the number of cell lines introduces variability—each line responds differently to culture conditions [24, 25]—thus challenging the interpretation of what is a disease phenotype or a cell line intrinsic behaviour. For disease modelling, standardised protocols and culture conditions are essential to safeguard from erroneous conclusions. Strategies like chimeroids, which combine multiple lines in the same organoid [8], offer a path forward. Future efforts should prioritise ensuring cellular homogeneity across cell lines prior to neural differentiation, potentially through pre‐patterning, and improving ethnic representation to enhance the generalizability and the translational relevance of findings.

The pursuit of tissue complexity *in vitro* requires a careful balance between expanding the potential of organoids to mimic *in vivo* development and avoiding the mistaken assumption that resemblance to *in vivo* structures implies full biological equivalence. The attribution of *in vivo* brain properties to neural organoids has led to unverified statements regarding the presence of consciousness or pain in organoids. On the other hand, the search for more comprehensive multi‐tissue organoids with cells from different germ layers interacting has enabled functional studies on neural development and provided insights into disease mechanisms. Complexity can be achieved via self‐assembly of multiple cell types from common progenitors [11, 12, 61, 62], organoid seeding with exogenous cell types [15], tissue build up from pre‐differentiated cell types [14], or enforced assembly of pre‐differentiated organoids into assembloids [4, 5, 47]. Given the challenge of ensuring the differentiation of multiple tissues from PSCs and their subsequent interaction, future research may focus on generating multi‐tissue organoids from a single progenitor population, balancing physiological relevance with reproducibility.

## Author contributions

IC and MAL researched the literature and wrote the manuscript. IC drew the Figures.

## Conflict of interest

M.A.L. is an inventor on patents related to cerebral organoids, with licensing agreements with Stem Cell Technologies, and is co‐founder of a:head bio.

## References

[1] Chiaradia I and Lancaster MA (2020) Brain organoids for the study of human neurobiology at the interface of in vitro and in vivo. Nat Neurosci 23, 1496–1508. 10.1038/s41593-020-00730-3 33139941

[2] Pașca SP , Arlotta P , Bateup HS , Camp JG , Cappello S , Gage FH , Knoblich JA , Kriegstein AR , Lancaster MA , Ming GL *et al*. (2022) A nomenclature consensus for nervous system organoids and assembloids. Nature 609, 907–910. 10.1038/s41586-022-05219-6 PMC10571504 36171373

[3] Smits LM , Reinhardt L , Reinhardt P , Glatza M , Monzel AS , Stanslowsky N , Rosato‐Siri MD , Zanon A , Antony PM , Bellmann J *et al*. (2019) Modeling Parkinson's disease in midbrain‐like organoids. NPJ Parkinsons Dis 5, 5. 10.1038/s41531-019-0078-4 PMC6450999 30963107

[4] Bagley JA , Reumann D , Bian S , Lévi‐Strauss J and Knoblich JA (2017) Fused cerebral organoids model interactions between brain regions. Nat Methods 14, 743–751. 10.1038/nmeth.4304 PMC5540177 28504681

[5] Birey F , Andersen J , Makinson CD , Islam S , Wei W , Huber N , Fan HC , Metzler KRC , Panagiotakos G , Thom N *et al*. (2017) Assembly of functionally integrated human forebrain spheroids. Nature 545, 54–59. 10.1038/nature22330 PMC5805137 28445465

[6] Chambers SM , Fasano CA , Papapetrou EP , Tomishima M , Sadelain M and Studer L (2009) Highly efficient neural conversion of human ES and iPS cells by dual inhibition of SMAD signaling. Nat Biotechnol 27, 275–280. 10.1038/nbt.1529 PMC2756723 19252484

[7] Shi Y , Kirwan P , Smith J , Robinson HPC and Livesey FJ (2012) Human cerebral cortex development from pluripotent stem cells to functional excitatory synapses. Nat Neurosci 15, 477–486. 10.1038/nn.3041 PMC3882590 22306606

[8] Antón‐Bolaños N , Faravelli I , Faits T , Andreadis S , Kastli R , Trattaro S , Adiconis X , Wei A , Sampath Kumar A , Di Bella DJ *et al*. (2024) Brain chimeroids reveal individual susceptibility to neurotoxic triggers. Nature 631, 142–149. 10.1038/s41586-024-07578-8 PMC11338177 38926573

[9] Li C , Fleck JS , Martins‐Costa C , Burkard TR , Themann J , Stuempflen M , Peer AM , Vertesy Á , Littleboy JB , Esk C *et al*. (2023) Single‐cell brain organoid screening identifies developmental defects in autism. Nature 621, 373–380. 10.1038/s41586-023-06473-y PMC10499611 37704762

[10] Wells MF , Nemesh J , Ghosh S , Mitchell JM , Salick MR , Mello CJ , Meyer D , Pietilainen O , Piccioni F , Guss EJ *et al*. (2023) Natural variation in gene expression and viral susceptibility revealed by neural progenitor cell villages. Cell Stem Cell 30, 312–332. 10.1016/j.stem.2023.01.010 PMC10581885 36796362

[11] Yin Y , Zhou W , Zhu J , Chen Z , Jiang L , Zhuang X , Chen J , Wei J , Lu X , Liu Y *et al*. (2025) Generation of self‐organized neuromusculoskeletal tri‐tissue organoids from human pluripotent stem cells. Stem Cells 32, 157–171. 10.1016/j.stem.2024.11.005 39657678

[12] Childs CJ , Poling HM , Chen K , Tsai YH , Wu A , Vallie A , Eiken MK , Huang S , Sweet CW , Schreiner R *et al*. (2025) Coordinated differentiation of human intestinal organoids with functional enteric neurons and vasculature. Cell Stem Cell 32, 640–651. 10.1016/j.stem.2025.02.007 PMC11973701 40043706

[13] Onesto MM , Kim J i and Pasca SP (2024) Assembloid models of cell‐cell interaction to study tissue and disease biology. Cell Stem Cell 31, 1563–1573. 10.1016/j.stem.2024.09.017 PMC12143640 39454582

[14] Abud EM , Ramirez RN , Martinez ES , Healy LM , Nguyen CHH , Newman SA , Yeromin AV , Scarfone VM , Marsh SE , Fimbres C *et al*. (2017) iPSC‐derived human microglia‐like cells to study neurological diseases. Neuron 94, 278–293. 10.1016/j.neuron.2017.03.042 PMC5482419 28426964

[15] Shi Y , Sun L , Wang MI , Liu JI , Zhong SI , Li R , Li P , Guo L , Fang A , ChenID R *et al*. (2020) Vascularized human cortical organoids (vOrganoids) model cortical development in vivo. 16 Human Genomic Variation 18, e3000705. 10.1371/journal.pbio.3000705 PMC7250475 32401820

[16] Sjöstedt E , Zhong W , Fagerberg L , Karlsson M , Mitsios N , Adori C , Oksvold P , Edfors F , Limiszewska A , Hikmet F *et al*. (2020) An atlas of the protein‐coding genes in the human, pig, and mouse brain. Science (1979) 367, eaay5947. 10.1126/science.aay5947 32139519

[17] Choudhury A , Aron S , Botigué LR , Sengupta D , Botha G , Bensellak T , Wells G , Kumuthini J , Shriner D , Fakim YJ *et al*. (2020) High‐depth African genomes inform human migration and health. Nature 586, 741–748. 10.1038/s41586-020-2859-7 PMC7759466 33116287

[18] Naidoo J , Hurrell T and Scholefield J (2024) The generation of human induced pluripotent stem cell lines from individuals of black African ancestry in South Africa. Stem Cell Res 81, 103534. 10.1016/j.scr.2024.103534 39146664

[19] Ghosh S , Nehme R and Barrett LE (2022) Greater genetic diversity is needed in human pluripotent stem cell models. Nat Commun 13, 1–7. 10.1038/s41467-022-34940-z PMC9701202 36435871

[20] Gurwitz D and Steeg R (2024) Enriching iPSC research diversity: harnessing human biobank collections for improved ethnic representation. Drug Dev Res 85, e22227. 10.1002/ddr.22227 38943497

[21] “Coriell Institute for Medical Research.”

[22] Montgomery RA , Tatapudi VS , Leffell MS and Zachary AA (2018) HLA in transplantation. Nat Rev Nephrol 14, 558–570. 10.1038/s41581-018-0039-x 29985463

[23] Fraga AM , Sukoyan M , Rajan P , Braga DP d AF , Iaconelli A , Franco JG , Borges E and Pereira LV (2011) Establishment of a Brazilian line of human embryonic stem cells in defined medium: implications for cell therapy in an ethnically diverse population. Cell Transplant 20, 431–440. 10.3727/096368910X522261 20719082

[24] Kilpinen H , Goncalves A , Leha A , Afzal V , Alasoo K , Ashford S , Bala S , Bensaddek D , Casale FP , Culley OJ *et al*. (2017) Common genetic variation drives molecular heterogeneity in human iPSCs. Nature 546, 370. 10.1038/nature22403 PMC5524171 28489815

[25] Amps K , Andrews PW , Anyfantis G , Armstrong L , Avery S , Baharvand H , Baker J , Baker D , Munoz MB , Beil S *et al*. (2011) Screening a large, ethnically diverse population of human embryonic stem cells identifies a chromosome 20 minimal amplicon that confers a growth advantage. Nat Biotechnol 29, 1132. 10.1038/nbt.2051 PMC3454460 22119741

[26] Pașca SP , Arlotta P , Bateup HS , Camp JG , Cappello S , Gage FH , Knoblich JA , Kriegstein AR , Lancaster MA , Ming G‐L *et al*. (2024) A framework for neural organoids, assembloids and transplantation studies. Nature 639, 315–320. 10.1038/s41586-024-08487-6 39653126

[27] He Z , Dony L , Fleck JS , Szałata A , Li KX , Slišković I , Lin H‐C , Santel M , Atamian A , Quadrato G *et al*. (2024) An integrated transcriptomic cell atlas of human neural organoids. Nature 635, 690–698. 10.1038/s41586-024-08172-8 PMC11578878 39567792

[28] Kanton S , Boyle MJ , He Z , Santel M , Weigert A , Sanchís‐Calleja F , Guijarro P , Sidow L , Fleck JS , Han D *et al*. (2019) Organoid single‐cell genomic atlas uncovers human‐specific features of brain development. Nature 574, 418–422. 10.1038/s41586-019-1654-9 31619793

[29] Fleck JS , Jansen SMJ , Wollny D , Zenk F , Seimiya M , Jain A , Okamoto R , Santel M , He Z , Camp JG *et al*. (2023) Inferring and perturbing cell fate regulomes in human brain organoids. Nature 621, 365–372. 10.1038/s41586-022-05279-8 PMC10499607 36198796

[30] Sawada T , Chater TE , Sasagawa Y , Yoshimura M , Fujimori‐Tonou N , Tanaka K , Benjamin KJM , Paquola ACM , Erwin JA , Goda Y *et al*. (2020) Developmental excitation‐inhibition imbalance underlying psychoses revealed by single‐cell analyses of discordant twins‐derived cerebral organoids. Mol Psychiatry 11, 2695–2711. 10.1038/s41380-020-0844-z PMC7577852 32764691

[31] Uzquiano A , Kedaigle AJ , Pigoni M , Paulsen B , Adiconis X , Kim K , Faits T , Nagaraja S , Antón‐Bolaños N , Gerhardinger C *et al*. (2022) Proper acquisition of cell class identity in organoids allows definition of fate specification programs of the human cerebral cortex. Cell 185, 3770–3788. 10.1016/j.cell.2022.09.010 PMC9990683 36179669

[32] Vértesy Á , Eichmüller OL , Naas J , Novatchkova M , Esk C , Balmaña M , Ladstaetter S , Bock C , von Haeseler A and Knoblich JA (2022) Gruffi: an algorithm for computational removal of stressed cells from brain organoid transcriptomic datasets. EMBO J 41, e111118. 10.15252/embj.2022111118 PMC9433936 35919947

[33] Pellegrini L , Bonfio C , Chadwick J , Begum F , Skehel M and Lancaster MA (2020) Human CNS barrier‐forming organoids with cerebrospinal fluid production. Science 369, eaaz5626. 10.1126/science.aaz5626 PMC7116154 32527923

[34] Kelava I , Chiaradia I , Pellegrini L , Kalinka AT and Lancaster MA (2022) Androgens increase excitatory neurogenic potential in human brain organoids. Nature 602, 112–116. 10.1038/s41586-021-04330-4 PMC7612328 35046577

[35] Velasco S , Kedaigle AJ , Simmons SK , Nash A , Rocha M , Quadrato G , Paulsen B , Nguyen L , Adiconis X , Regev A *et al*. (2019) Individual brain organoids reproducibly form cell diversity of the human cerebral cortex. Nature 570, 523–527. 10.1038/s41586-019-1289-x PMC6906116 31168097

[36] Bhaduri A , Andrews MG , Mancia Leon W , Jung D , Shin D , Allen D , Jung D , Schmunk G , Haeussler M , Salma J *et al*. (2020) Cell stress in cortical organoids impairs molecular subtype specification. Nature 578, 142–148. 10.1038/s41586-020-1962-0 PMC7433012 31996853

[37] Paulsen B , Velasco S , Kedaigle AJ , Pigoni M , Quadrato G , Deo AJ , Adiconis X , Uzquiano A , Sartore R , Yang SM *et al*. (2022) Autism genes converge on asynchronous development of shared neuron classes. Nature 602, 268–273. 10.1038/s41586-021-04358-6 PMC8852827 35110736

[38] Khan TA , Revah O , Gordon A , Yoon SJ , Krawisz AK , Goold C , Sun Y , Kim CH , Tian Y , Li MY *et al*. (2020) Neuronal defects in a human cellular model of 22q11.2 deletion syndrome. Nat Med 26, 1888–1898. 10.1038/s41591-020-1043-9 PMC8525897 32989314

[39] Sloan SA , Darmanis S , Huber N , Khan TA , Birey F , Caneda C , Reimer R , Quake SR , Barres BA and Paşca SP (2017) Human astrocyte maturation captured in 3D cerebral cortical spheroids derived from pluripotent stem cells. Neuron 95, 779–790. 10.1016/j.neuron.2017.07.035 PMC5890820 28817799

[40] Trujillo CA , Gao R , Negraes PD , Gu J , Buchanan J , Preissl S , Wang A , Wu W , Haddad GG , Chaim IA *et al*. (2019) Complex oscillatory waves emerging from cortical organoids model early human brain network development. Cell Stem Cell 25, 558–569. 10.1016/j.stem.2019.08.002 PMC6778040 31474560

[41] Yoon SJ , Elahi LS , Pașca AM , Marton RM , Gordon A , Revah O , Miura Y , Walczak EM , Holdgate GM , Fan HC *et al*. (2019) Reliability of human cortical organoid generation. Nat Methods 16, 75–78. 10.1038/s41592-018-0255-0 PMC6677388 30573846

[42] Bowles KR , Silva MC , Whitney K , Bertucci T , Berlind JE , Lai JD , Garza JC , Boles NC , Mahali S , Strang KH *et al*. (2021) ELAVL4, splicing, and glutamatergic dysfunction precede neuron loss in MAPT mutation cerebral organoids. Cell 184, 4547–4563. 10.1016/j.cell.2021.07.003 PMC8635409 34314701

[43] Qian X , Su Y , Adam CD , Deutschmann AU , Pather SR , Goldberg EM , Su K , Li S , Lu L , Jacob F *et al*. (2020) Sliced human cortical organoids for modeling distinct cortical layer formation. Cell Stem Cell 26, 766–781. 10.1016/j.stem.2020.02.002 PMC7366517 32142682

[44] Miura Y , Li MY , Birey F , Ikeda K , Revah O , Thete MV , Park JY , Puno A , Lee SH , Porteus MH *et al*. (2020) Generation of human striatal organoids and cortico‐striatal assembloids from human pluripotent stem cells. Nat Biotechnol 38, 1421–1430. 10.1038/s41587-020-00763-w PMC9042317 33273741

[45] Samarasinghe RA , Miranda OA , Buth JE , Mitchell S , Ferando I , Watanabe M , Allison TF , Kurdian A , Fotion NN , Gandal MJ *et al*. (2021) Identification of neural oscillations and epileptiform changes in human brain organoids. Nat Neurosci 24, 1488–1500. 10.1038/s41593-021-00906-5 PMC9070733 34426698

[46] Huang WK , Wong SZH , Pather SR , Nguyen PTT , Zhang F , Zhang DY , Zhang Z , Lu L , Fang W , Chen L *et al*. (2021) Generation of hypothalamic arcuate organoids from human induced pluripotent stem cells. Cell Stem Cell 28, 1657–1670. 10.1016/j.stem.2021.04.006 PMC8419002 33961804

[47] Xiang Y , Tanaka Y , Cakir B , Patterson B , Kim KY , Sun P , Kang YJ , Zhong M , Liu X , Patra P *et al*. (2019) hESC‐derived thalamic organoids form reciprocal projections when fused with cortical organoids. Cell Stem Cell 24, 487–497. 10.1016/j.stem.2018.12.015 PMC6853597 30799279

[48] Fiorenzano A , Sozzi E , Birtele M , Kajtez J , Giacomoni J , Nilsson F , Bruzelius A , Sharma Y , Zhang Y , Mattsson B *et al*. (2021) Single‐cell transcriptomics captures features of human midbrain development and dopamine neuron diversity in brain organoids. Nat Commun 12, 7302. 10.1038/s41467-021-27464-5 PMC8674361 34911939

[49] Atamian A , Birtele M , Hosseini N , Nguyen T , Seth A , Del Dosso A , Paul S , Tedeschi N , Taylor R , Coba MP *et al*. (2024) Human cerebellar organoids with functional Purkinje cells. Cell Stem Cell 31, 39–51. 10.1016/j.stem.2023.11.013 PMC11417151 38181749

[50] Andersen J , Revah O , Miura Y , Thom N , Amin ND , Kelley KW , Singh M , Chen X , Thete MV , Walczak EM *et al*. (2020) Generation of functional human 3D Cortico‐motor Assembloids. Cell 183, 1913–1929. 10.1016/j.cell.2020.11.017 PMC8711252 33333020

[51] Marton RM , Miura Y , Sloan SA , Li Q , Revah O , Levy RJ , Huguenard JR and Pașca SP (2019) Differentiation and maturation of oligodendrocytes in human three‐dimensional neural cultures. Nat Neurosci 22, 484–491. 10.1038/s41593-018-0316-9 PMC6788758 30692691

[52] Cuomo ASE , Seaton DD , McCarthy DJ , Martinez I , Bonder MJ , Garcia‐Bernardo J , Amatya S , Madrigal P , Isaacson A , Buettner F *et al*. (2020) Single‐cell RNA‐sequencing of differentiating iPS cells reveals dynamic genetic effects on gene expression. Nat Commun 11, 1–14. 10.1038/s41467-020-14457-z PMC7010688 32041960

[53] Jerber J , Seaton DD , Cuomo ASE , Kumasaka N , Haldane J , Steer J , Patel M , Pearce D , Andersson M , Bonder MJ *et al*. (2021) Population‐scale single‐cell RNA‐seq profiling across dopaminergic neuron differentiation. Nat Genet 53, 304. 10.1038/s41588-021-00801-6 PMC7610897 33664506

[54] van der Wijst MGP , de Vries DH , Groot HE , Trynka G , Hon CC , Bonder MJ , Stegle O , Nawijn MC , Idaghdour Y , van der Harst P *et al*. (2020) The single‐cell eQTLGen consortium. elife 9, e52155. 10.7554/eLife.52155 PMC7077978 32149610

[55] Neavin DR , Steinmann AM , Farbehi N , Chiu HS , Daniszewski MS , Arora H , Bermudez Y , Moutinho C , Chan CL , Bax M *et al*. (2023) A village in a dish model system for population‐scale hiPSC studies. Nat Commun 14, 1–12. 10.1038/s41467-023-38704-1 PMC10256711 37296104

[56] Cederquist GY , Tchieu J , Callahan SJ , Ramnarine K , Ryan S , Zhang C , Rittenhouse C , Zeltner N , Chung SY , Zhou T *et al*. (2020) A multiplex human pluripotent stem cell platform defines molecular and functional subclasses of autism‐related genes. Cell Stem Cell 27, 35–49. 10.1016/j.stem.2020.06.004 PMC7376579 32619517

[57] Caporale N , Castaldi D , Rigoli MT , Cheroni C , Valenti A , Stucchi S , Lessi M , Bulgheresi D , Trattaro S , Pezzali M *et al*. (2024) Multiplexing cortical brain organoids for the longitudinal dissection of developmental traits at single‐cell resolution. Nat Methods 22, 358–370. 10.1038/s41592-024-02555-5 PMC11810796 39653820

[58] He Z , Maynard A , Jain A , Gerber T , Petri R , Lin HC , Santel M , Ly K , Dupré JS , Sidow L *et al*. (2022) Lineage Recording in Human Cerebral Organoids. Springer US, New York, NY. 10.1038/s41592-021-01344-8 PMC8748197 34969984

[59] Gouti M , Tsakiridis A , Wymeersch FJ , Huang Y and Kleinjung J (2014) In vitro generation of Neuromesodermal progenitors reveals distinct roles for Wnt Signalling in the specification of spinal Cord and paraxial mesoderm identity. PLoS Biol 12, 1001937. 10.1371/journal.pbio.1001937 PMC4144800 25157815

[60] Tzouanacou E , Wegener A , Wymeersch FJ , Wilson V and Nicolas JF (2009) Redefining the progression of lineage segregations during mammalian embryogenesis by clonal analysis. Dev Cell 17, 365–376. 10.1016/j.devcel.2009.08.002 19758561

[61] Lancaster MA , Renner M , Martin CA , Wenzel D , Bicknell LS , Hurles ME , Homfray T , Penninger JM , Jackson AP and Knoblich JA (2013) Cerebral organoids model human brain development and microcephaly. Nature 501, 373–379. 10.1038/nature12517 PMC3817409 23995685

[62] Quadrato G , Nguyen T , Macosko EZ , Sherwood JL , Yang SM , Berger DR , Maria N , Scholvin J , Goldman M , Kinney JP *et al*. (2017) Cell diversity and network dynamics in photosensitive human brain organoids. Nature 545, 48–53. 10.1038/nature22047 PMC5659341 28445462

[63] Guglielmi L , Lloyd‐Davies‐Sánchez D , Martínez JG and Lancaster MA (2024) “A minimally guided organoid model for cross‐species comparisons of cerebellar development.” bioRxiv, 2024.10.02.616236.

[64] Pang W , Zhu J , Yang K , Zhu X , Zhou W , Jiang L , Zhuang X , Liu Y , Wei J , Lu X *et al*. (2024) Generation of human region‐specific brain organoids with medullary spinal trigeminal nuclei. Cell Stem Cell 31, 1501–1512. 10.1016/j.stem.2024.08.004 39208804

[65] Lee JH , Shin H , Shaker MR , Kim HJ , Park SH , Kim JH , Lee N , Kang M , Cho S , Kwak TH *et al*. (2022) Production of human spinal‐cord organoids recapitulating neural‐tube morphogenesis. Nat Biomed Eng 6, 435–448. 10.1038/s41551-022-00868-4 35347276

[66] Eicher AK , Kechele DO , Sundaram N , Berns HM , Poling HM , Haines LE , Sanchez JG , Kishimoto K , Krishnamurthy M , Han L *et al*. (2022) Functional human gastrointestinal organoids can be engineered from three primary germ layers derived separately from pluripotent stem cells. Cell Stem Cell 29, 36–51. 10.1016/j.stem.2021.10.010 PMC8741755 34856121

[67] Ormel PR , de Vieira Sá R , van Bodegraven EJ , Karst H , Harschnitz O , Sneeboer MAM , Johansen LE , van Dijk RE , Scheefhals N , van Berdenis Berlekom A *et al*. (2018) Microglia innately develop within cerebral organoids. Nat Commun 9, 4167. 10.1038/s41467-018-06684-2 PMC6177485 30301888

[68] Cakir B , Xiang Y , Tanaka Y , Kural MH , Parent M , Kang Y‐J , Chapeton K , Patterson B , Yuan Y , He C‐S *et al*. (2020) “Development of human brain organoids with functional vascular‐like system HHS public access.” 10.1038/s41592-019-0586-5 PMC6918722 31591580

[69] Sun XY , Ju XC , Li Y , Zeng PM , Wu J , Zhou YY , Shen LB , Dong J , Chen YJ and Luo ZG (2022) Generation of vascularized brain organoids to study neurovascular interactions. elife 11, e76707. 10.7554/eLife.76707 PMC9246368 35506651

[70] Takebe T , Enomura M , Yoshizawa E , Kimura M , Koike H , Ueno Y , Matsuzaki T , Yamazaki T , Toyohara T , Osafune K *et al*. (2015) Vascularized and complex organ buds from diverse tissues via mesenchymal cell‐driven condensation. Cell Stem Cell 16, 556–565. 10.1016/j.stem.2015.03.004 25891906

[71] Stiles J and Jernigan TL (2010) The basics of brain development. Neuropsychol Rev 20, 327–348. 10.1007/s11065-010-9148-4 PMC2989000 21042938

[72] Simeone A , Acampora D , Mallamaci A , Stornaiuolo A , D'Apice MR , Nigro V and Boncinelli E (1993) A vertebrate gene related to orthodenticle contains a homeodomain of the bicoid class and demarcates anterior neuroectoderm in the gastrulating mouse embryo. EMBO J 12, 2735–2747. 10.1002/j.1460-2075.1993.tb05935.x PMC413524 8101484

[73] Gaspard N , Bouschet T , Hourez R , Dimidschstein J , Naeije G , Van Den Ameele J , Espuny‐Camacho I , Herpoel A , Passante L , Schiffmann SN *et al*. (2008) An intrinsic mechanism of corticogenesis from embryonic stem cells. Nature 455, 351–357. 10.1038/nature07287 18716623

[74] Roth JG , Brunel LG , Huang MS , Liu Y , Cai B , Sinha S , Yang F , Pașca SP , Shin S and Heilshorn SC (2023) Spatially controlled construction of assembloids using bioprinting. Nat Commun 14, 1–14. 10.1038/s41467-023-40006-5 PMC10356773 37468483

[75] Kim J , Imaizumi K , Jurjuț O , Kelley KW , Wang D , Thete MV , Hudacova Z , Amin ND , Levy RJ , Scherrer G *et al*. (2025) Human assembloid model of the ascending neural sensory pathway. Nature 642, 143–153. 10.1038/s41586-025-08808-3 PMC12137141 40205039

[76] Bosone C , Castaldi D , Burkard TR , Guzman SJ , Wyatt T , Cheroni C , Caporale N , Bajaj S , Bagley JA , Li C *et al*. (2024) A polarized FGF8 source specifies frontotemporal signatures in spatially oriented cell populations of cortical assembloids. Nat Methods 21, 2147–2159. 10.1038/s41592-024-02412-5 PMC11541204 39294368

[77] Miura Y , Kim J , Jurjuț O , Kelley KW , Yang X , Chen X , Thete MV , Revah O , Cui B , Pachitariu M *et al*. (2024) “Assembloid model to study loop circuits of the human nervous system.” bioRxiv.

[78] Amadei G , Handford CE , Qiu C , De Jonghe J , Greenfeld H , Tran M , Martin BK , Chen DY , Aguilera‐Castrejon A , Hanna JH *et al*. (2022) Embryo model completes gastrulation to neurulation and organogenesis. Nature 610, 143–153. 10.1038/s41586-022-05246-3 PMC9534772 36007540

[79] Tarazi S , Aguilera‐Castrejon A , Joubran C , Ghanem N , Ashouokhi S , Roncato F , Wildschutz E , Haddad M , Oldak B , Gomez‐Cesar E *et al*. (2022) Post‐gastrulation synthetic embryos generated ex utero from mouse naive ESCs. Cell 185, 3290–3306. 10.1016/j.cell.2022.07.028 PMC9439721 35988542

[80] Park ES , Georgescu A and Huh D (2019) Organoids‐on‐a‐chip. Science (1979) 364, 960–965. 10.1126/science.aaw7894 PMC7764943 31171693

[81] Eiraku M , Watanabe K , Matsuo‐Takasaki M , Kawada M , Yonemura S , Matsumura M , Wataya T , Nishiyama A , Muguruma K and Sasai Y (2008) Self‐organized formation of polarized cortical tissues from ESCs and its active manipulation by extrinsic signals. Cell Stem Cell 3, 519–532. 10.1016/j.stem.2008.09.002 18983967

[82] Paşca AM , Sloan SA , Clarke LE , Tian Y , Makinson CD , Huber N , Kim CH , Park JY , O'Rourke NA , Nguyen KD *et al*. (2015) Functional cortical neurons and astrocytes from human pluripotent stem cells in 3D culture. Nat Methods 12, 671–678. 10.1038/nmeth.3415 PMC4489980 26005811

[83] Revah O , Gore F , Kelley KW , Andersen J , Sakai N , Chen X , Li MY , Birey F , Yang X , Saw NL *et al*. (2022) Maturation and circuit integration of transplanted human cortical organoids. Nature 610, 319–326. 10.1038/s41586-022-05277-w PMC9556304 36224417

[84] Giandomenico SL , Mierau SB , Gibbons GM , Wenger LMD , Masullo L , Sit T , Sutcliffe M , Boulanger J , Tripodi M , Derivery E *et al*. (2019) Cerebral organoids at the air‐liquid interface generate diverse nerve tracts with functional output. Nat Neurosci 22, 669–679. 10.1038/s41593-019-0350-2 PMC6436729 30886407

[85] Qian X , Nguyen HN , Song MM , Hadiono C , Ogden SC , Hammack C , Yao B , Hamersky GR , Jacob F , Zhong C *et al*. (2016) Brain‐region‐specific organoids using mini‐bioreactors for modeling ZIKV exposure. Cell 165, 1238–1254. 10.1016/j.cell.2016.04.032 PMC4900885 27118425

[86] Zhou T , Tan L , Cederquist GY , Fan Y , Hartley BJ , Mukherjee S , Tomishima M , Brennand KJ , Zhang Q , Schwartz RE *et al*. (2017) High‐content screening in hPSC‐neural progenitors identifies drug candidates that inhibit Zika virus infection in fetal‐like organoids and adult brain. Cell Stem Cell 21, 274–283. 10.1016/j.stem.2017.06.017 PMC5553280 28736217

[87] Li Y , Muffat J , Omer A , Bosch I , Lancaster MA , Sur M , Gehrke L , Knoblich JA and Jaenisch R (2017) Induction of expansion and folding in human cerebral organoids. Cell Stem Cell 20, 385–396. 10.1016/j.stem.2016.11.017 PMC6461394 28041895

[88] Mariani J , Coppola G , Zhang P , Abyzov A , Provini L , Tomasini L , Amenduni M , Szekely A , Palejev D , Wilson M *et al*. (2015) FOXG1‐dependent dysregulation of GABA/glutamate neuron differentiation in autism Spectrum disorders. Cell 162, 375–390. 10.1016/j.cell.2015.06.034 PMC4519016 26186191

[89] Paşca SP , Portmann T , Voineagu I , Yazawa M , Shcheglovitov A , Paşca AM , Cord B , Palmer TD , Chikahisa S , Nishino S *et al*. (2011) Using iPSC‐derived neurons to uncover cellular phenotypes associated with Timothy syndrome. Nat Med 17, 1657–1662. 10.1038/nm.2576 PMC3517299 22120178

[90] Gomes AR , Fernandes TG , Vaz SH , Silva TP , Bekman EP , Xapelli S , Duarte S , Ghazvini M , Gribnau J , Muotri AR *et al*. (2020) Modeling rett syndrome with human patient‐specific forebrain organoids. Front Cell Dev Biol 8, 610427. 10.3389/fcell.2020.610427 PMC7758289 33363173

